# Specific Changes of Exocarp and Mesocarp Occurring during Softening Differently Affect Firmness in Melting (MF) and Non Melting Flesh (NMF) Fruits

**DOI:** 10.1371/journal.pone.0145341

**Published:** 2015-12-28

**Authors:** E. Onelli, A. Ghiani, R. Gentili, S. Serra, S. Musacchi, S. Citterio

**Affiliations:** 1 Department of Biosciences, University of Milan, Via Celoria 26, 20133, Milano, Italy; 2 Department of Earth and Environmental Sciences, University of Milano-Bicocca, Piazza della Scienza n. 1, 20126, Milan, Italy; 3 Department of Horticulture, Washington State University, TFREC, Wenatchee, 98801, WA, United States of America; University of Minho, PORTUGAL

## Abstract

Melting (MF) and non melting flesh (NMF) peaches differ in their final texture and firmness. Their specific characteristics are achieved by softening process and directly dictate fruit shelf life and quality. Softening is influenced by various mechanisms including cell wall reorganization and water loss. In this work, the biomechanical properties of MF Spring Crest’s and NMF Oro A’s exocarp and mesocarp along with the amount and localization of hydroxycinnamic acids and flavonoids were investigated during fruit ripening and post-harvest. The objective was to better understand the role played by water loss and cell wall reorganization in peach softening. Results showed that in ripe Spring Crest, where both cell turgor loss and cell wall dismantling occurred, mesocarp had a little role in the fruit reaction to compression and probe penetration response was almost exclusively ascribed to the epidermis which functioned as a mechanical support to the pulp. In ripe Oro A’s fruit, where cell wall disassembly did not occur and the loss of cell turgor was observed only in mesocarp, the contribution of exocarp to fruit firmness was consistent but relatively lower than that of mesocarp, suggesting that in addition to cell turgor, the integrity of cell wall played a key role in maintaining NMF fruit firmness. The analysis of phenols suggested that permeability and firmness of epidermis were associated with the presence of flavonoids and hydroxycinnamic acids.

## Introduction

Fruit softening is one of the main physiological processes occurring during ripening of many fleshy fruits. It seems to be mainly determined by two integrated mechanisms: cell wall modification and water/turgor loss leading to textural changes and loss of firmness, respectively [1, 2].

Wall modification has been widely investigated in different fruits including peach; it is a complex process due to both degradation and synthesis activities involving a high number of polysaccharides, proteins and metabolites [3, 4]. For a long time, the polygalacturonase-catalysed depolymerization of pectins has been considered the principal mechanism contributing to fruit softening as the decline in fruit firmness typically coincides with the dissolution of the middle lamella, resulting in a reduction of intercellular adhesion [5]. However, this hypothesis has been refuted through reverse genetics studies in tomato [6] and only recently Saladiè and co-workers [1] pointed out other ripening-related physiological processes, such as cellular turgor and morphology, as critical players in fruit softening. Specifically, these authors identified cuticle as a determinant factor regulating water status and working as physical support. Cuticle is, in fact, accounted as the main cell structure involved in limiting transpiration and maintaining fruit integrity avoiding microbial infection [7–9]. It is also an important player in the postharvest quality of fruits [10]. The permeability of cuticle was correlated with its degree of wax coverage, thickness and composition/assembly of chemical compounds [11]. Indeed, analyses of lipid composition on firm-fleshed peach mutant showed an important correlation between fatty acid chemistry and fruit firmness supporting the role of cuticle in water loss regulation [12]. In addition to lipids, polysaccharides and phenolic compounds can influence cuticle permeability and cell wall mechanical properties [13, 14]. Hydroxycinnamic acids (HC) and flavonoids can be present in the cuticle as free compounds trapped in the matrix and/or bound to epidermis cell wall or cutin component by ester or ether bonds [15]. Specifically, ferulic (FA) and coumaric (CA) acids are contained in the cell wall of many plants and are ester-linked mainly to hemicellulosic arabinoxylans [16]. Cell wall polysaccharides-bound FA can undergo a peroxidase-catalyzed coupling reaction to produce diferulic acid (DFA), which cross-links arabinoxylans or more in general cell wall components. Fry et al. [17] showed that the cross-linkage of polysaccharides by DFA-bridges contributes to wall assembly, promoting tissue cohesion and restricting cell wall extensibility. Like DFA, FA itself may decrease the cell wall extensibility by interfering with enzymatic degradation of cell wall polysaccharides [17]. For instance, changes in the amounts of wall-bound DFA and FA in oat and rice coleoptiles were closely correlated with their cell wall extensibility [18, 19]. Moreover HC acid derivatives were shown to be tightly bound to the epicuticular waxes of peach, olive tree, tomato and apple leaves [15, 20] and to cuticle of different fruits [21, 22]. They were considered structural constituents having a role in the cross-linking of cell wall polymers and thus in determining biomechanical properties and permeability of the plant organ surface [20, 23]. Also flavonoids serve as structural elements influencing the mechanical properties of the epidermal cell layer of fruits. In addition, they seem to modulate tomato cuticle water transpiration by affecting wax synthesis and deposition [24, 25].

Peach cultivar are classified as melting flesh (MF) and non-melting flesh (NMF), according to fruit firmness and texture characteristics.

Ripe NMF fruits are known for maintaining their firmness and are traditionally grown for canning purposes, whereas MF peaches are usually grown for the fresh market as the fruit becomes soft and shows suitable quality characteristics (red coloration, acidity, and aroma) lacking in NMF fruit (Fig A in S1 File). Moreover, MF peaches show a propensity to mechanical damage and decay during handling [26]

In MF fruit, softening occurs in two stages. Tissue firmness decreases slowly and progressively in the first stage, whereas, in the second one, the drop of tissue firmness is rapid. This last phase of softening is called ‘melting’ stage. Differently from MF, NMF peaches lack the final melting phase of softening. The different final grade of firmness and texture of MF and NMF fruits is attributed to differences in both water/turgor loss and endo-PG activity [2]. Specifically, it was recently suggested that endo-PG function is needed to achieve melting flesh texture, and that the change of symplast/apoplast water status seems to be the main process through which fruit regulates its firmness [2]. Nevertheless in NMF pericarp, cell walls remain adherent and their stiffness could contribute to the fruit firmness. In order to verify the real contribution of pericarp cell wall dismantling to firmness loss, in this work we investigated the changes in biomechanical properties of MF and NMF fruits during ripening. In addition, changes in biomechanical properties were correlated to HC acid and flavonoid content and localization during softening in order to delve into their possible role in the regulation of water loss and firmness of epidermis cell wall/cuticle.

## Materials and Methods

### Plant material and fruit ripening assessment

A collection field of peach (*Prunus persica* L. Batsch) trees grafted on GF677 rootstock was planted in 2012 with a planting density of 4.0 m x 1.5 m. Trees were trained to spindle and irrigated by dripping system. The field trail was located (44°32'49.7"N 11°23'10.3"E) at the experimental station of the Department of Agricultural Sciences, University of Bologna (Cadriano, Italy).

Two peach cultivars: Spring Crest (MF), and Oro A (NMF) were monitored during the growing season on the tree to define the ripening stages. Starting from 52 days after full bloom (DAB), for both cultivars, Index of Absorbance Difference (I_AD_) was measured by DA-Meter (TR, Forlì, Italy) on-tree fruit once a week and more frequently close to the harvest. I_DA_ is correlated to the chlorophyll-a content, fruit quality traits and to the transcription of some ripening-related genes [27, 28]. A subsample of them was used to measure fruit ethylene production. Immediately after harvest, fruit not utilized for ethylene measurement were sealed with a specific wax on the stalk region to avoid water loss before further analyses.

Fruits were harvested in two picking times in relation to the ethylene production: pre-climacteric (pre-C), post-climacteric (post-C) and selected as much homogenous as possible within the most representative I_AD_ class for each stage. Ethylene production was measured in static condition sealing each fruit in a 1L jar as described in Ziosi et al. [28].

For Spring Crest (SC), the pre-C stage corresponded to 79 DAB and all fruit sampled for further analysis belonged to the 0.80–1.00 I_AD_ class with an average ethylene production of 0.51 nL h^-1^g^-1^FW. SC’s post-climateric stage was at 91 DAB and the fruit ripening class representative of the stage was 0.00–0.10 I_AD_ with an average ethylene production of 9.15 nL h^-1^g^-1^FW. A subsample of post-C SC fruit was stored at 25°C for 3 days to assess the ripening in post-harvest (post-H) stage. After storage, they produced ethylene with an average value of 52.07 nL h^-1^g^-1^FW.

For Oro A the same procedure was adopted. The pre-climacteric stage corresponded to 99 DAB and fruit belonging to the 1.50–2.00 I_AD_ class showed an average ethylene production of 0.60 nL h^-1^g^-1^FW. The post-C stage was at 105 DAB and the fruit ripening class representative of the stage was 0.00–0.06 I_AD_ with an average ethylene production of 19.23 nL h^-1^g^-1^FW. Post-H fruit after storage showed an average ethylene production of 105.56 nL h^-1^g^-1^FW.

### Biomechanical analyses by dynamometer

Biomechanical analyses were performed by a Dynamometer Texture Analyzer TA HD Plus Stable MicroSystem (ENCO) with a flat-end cylindrical probe (6.5 mm diameter section) and equipped with a 5 kg load cell. Six NMF and six MF peach fruits were analysed for each ripening stage. Four penetrations in each whole fruit and two penetrations in the same peeled fruit were carried out. All the fruits were peeled by removing 1 mm skin with a special knife. About the same number of cell layers was removed from SC and Oro A fruits at each stage (35–40 layers at pre-C and 1–2 layers less at post-C and post-H stages). In order to make 10–13 mm deep penetration into the fruit tissues, 50 kg strength at a constant rate (0.04 mm s^-1^) for a fixed time (250 s) was applied. Force-strain curves were recorded in real time during the analysis. Dynamometer recorded 400 measures s^-1^ for each parameter considered. Data were statistically analyzed with the Statgraphics plus program for Windows (version 5.0, Manugistic, Maryland USA).

### Microscopy

For microscopy analysis, 2 mm blocks of exocarp and mesocarp were collected from 4 different SC and Oro A fruits for each ripening stage as previously described [2].

Samples were infused with 20% and 30% sucrose in cacodylate buffer (0.1 M pH 6.9), as cryoprotectant for 2 days. Afterwards, samples were immersed in TFM (Tissue Freezing Medium, EMS), frozen in liquid nitrogen and stored at -20°C. Eight μm sections were obtained by cryostat pre-cooled at -20°C (Leica CM1800, Germany). The slides were stored at -20°C.

Blue/green autofluorescence (BGF) was observed on fresh frozen sections before and after a treatment with 0.1% ammonia (NH_3_). Sections were examined by a Zeiss Axioplan microscope connected to a video camera (Media Cybernetics, Silver Spring, Maryland). In order to distinguish between blue and green autofluorescent sections were also observed by a Leica TCS SP5 confocal microscope (Leica Microsystems, GmbH, Wetzlar, Germany).

For flavonoids observation, sections were stained with 0.5% diphenylboric acid 2-aminoethyl ester (NA) in sample buffer (100 mM K_2_HPO_4_/NaH_2_PO_4_, pH 6.8, 1% NaCl) and examined by the conventional and confocal microscope.

### Content of phenolic compounds

To quantify phenolic compounds in the peach exocarp, frozen fruit samples were ground in liquid nitrogen and then incubated in 80% aqueous methanol acidified with 0.1% HCl for 3h at room temperature. The mixture was then centrifuged at 12,000 g for 20 min at 4°C. The supernatant (S1), containing free-soluble polyphenols, was collected. The resulting residue (P1) was used to extract cell wall-bounded phenolic compounds. P1 was hydrolyzed with 2 N NaOH, stirring it for 1h. The reaction mixture was then acidified with 7.2 N HCl. The released phenolic compounds were extracted with ethylacetate (2 times). The organic layers were combined and evaporated at 45°C under vacuum. The residue was dissolved in 25 mL methanol­water (80:20 v/v) with 0.1% HCl.

The amount of free-soluble and wall-bound phenolic compounds was estimated by using the Folin-Ciocalteau method. A calibration curve was prepared using gallic acid solution. Phenolics content was expressed as milligrams of gallic acid equivalent (mg GAE) per 100 g of fresh weight (FW). All samples were prepared in triplicate.

The amount of free-soluble and wall-bound flavonoids was assessed by aluminum chloride colorimetric assay [29]. Quercitin was used as standard for the construction of the calibration curve and the concentrations were expressed as milligrams of quercitin equivalents per 100 g of fresh weight. All samples were analysed in triplicate. HC content was calculated subtracting flavonoid content from total phenolic content.

### Extraction of total RNA and RT-PCR experiments

In order to study the expression of Endo-PG and CHS genes, total RNA was extracted from frozen fruit samples following the protocol of Lester et al. [30] and used for reverse transcription-PCR (RT-PCR) experiments. RNA yield and integrity were checked by UV absorption spectra and electrophoresis in a 1% agarose gel, respectively.

For cDNA synthesis, a GeneAmp EZ rTth PCR kit from Applied Biosystems was used according to the manufacturer’s instructions. The cDNA was eluted 10 times and used as template for PCR analysis with Endo-PG (GenBank accession no. DQ340809) and CHS (GenBank accession no. XM_007222963) specific primers (EndoPG forward: 5’-TCTTCGCGATGTGGTGTTCA-3’, EndoPG reverse: 5’- GCCATCGGTGTTAGGGCTAT-3’; CHS forward: 5'-TCAGCTCACTAAGCTCTTGGGC-3', CHS reverse: 5’-TCAAGCATTCAAGCCCACGC-3’).

The 50 μL reaction mixture contained 10 pmol of each specific forward and reverse primer, 1X PCR buffer (50 mM KCl, 1.5 mM MgCl_2_, 10 mM TRIS-HCl), 1 mM dNTPs, and 1 U Taq DNA polymerase (Pharmacia Biotech, USA). The following cycling conditions ensured optimal primer selectivity: after a first step of 3 min at 94°C, 30 cycles of 94°C for 40 s, optimal annealing temperature (60°C) for 1 min, and 72°C for 1 min were performed. Comparison of the sample signal intensities was carried out using the Gel Doc 2000 image analysis system (BioRad, Milan, Italy).

### Extraction of proteins and Western blot analyses

Proteins were extracted from frozen fruit samples in extraction buffer (2% SDS, 60 mM DTT, 20% glycerol, 40 mM Tris-HCl ph 8.8). After 8 min boiling, samples were centrifuged at 7,500 *xg* for 15 minutes and then rinsed for 1 h in 3 volumes acetone and 20 mM DTT. After dehydration, pellet was resuspendend in 1% SDS. SDS-PAGE was performed as described by Laemmli [31]. After gel electrophoresis, proteins were blotted onto a 0.45 μm nitrocellulose membrane by a Trans-Blot cell (Bio-Rad, Milano, Italy) containing transfer buffer (25 mM Tris, 192 mM glycine and 20% (v/v) methanol, pH 8.3). Membranes were blocked with 5% (w/v) non-fat dry milk powder in TBS-T (20 mM Tris, 150 mM NaCl and 0.05% (v/v) Tween 20, pH 7.5) for 1 h and then incubated with anti-endo-PG (1 μg/mL) and anti-CHS (0.4 μg/mL) for 1h in TBS-T. After three rinses in TBS-T for 5 min each, membranes were soaked for 1 h with alkaline phosphatase-conjugated goat anti-rabbit IgG (Sigma-Aldrich) diluted 1:15000 in TBS-T. Membranes were then washed three more times with TBS-T. Immunoreactive bands were detected by Sigma Fast BCIP/NBT as an alkaline phosphatase substrate according to the manufacturer’s protocol.

## Results

### Characterization of peach fruits during maturation

**Fig 1:**
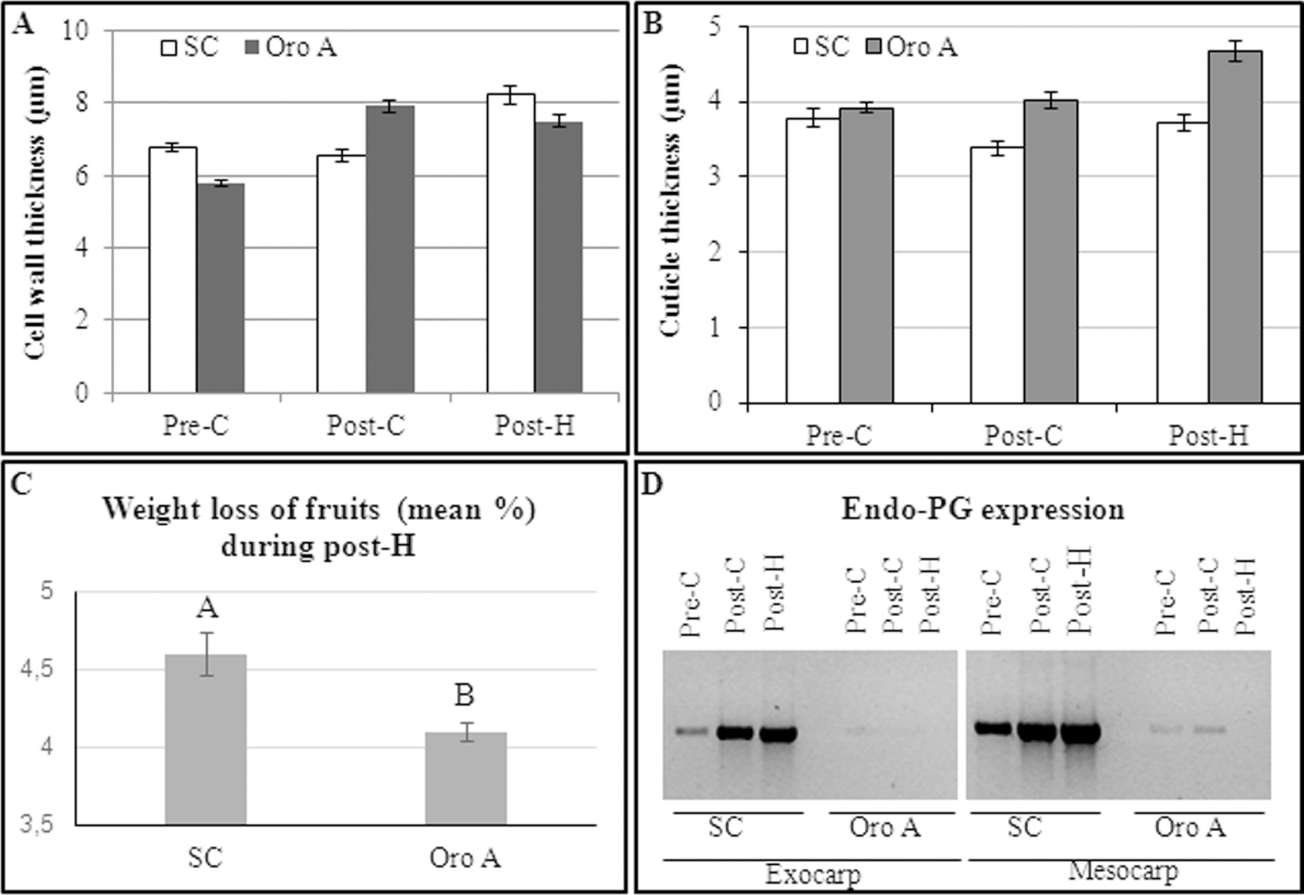
Fruit external epidermal cell wall (A) and cuticle (B) thickness, fruit weigh loss (mean percentage) during post-H (C) and endopolygalacturonase expression (EndoPG, D) in Oro A and SC cultivar. Different letters indicate significant differences (P<0.05).

SC (MF) and Oro A (NMF) fruits were collected at three ripening stages: pre–C, post-C and 2 days of post-H. The stages were defined on the basis of I_AD_, ethylene production (Table 1) and endo-PG synthesis (Fig 1). The thickness of fruit external epidermal cell wall and cuticle during ripening and the water loss during post-harvest, through the evaluation of fruit weight change, were also determined (Fig 1).

**Table 1:** Characteristics of Oro A and Spring Crest fruits at the selected ripening (pre- and post-climacteric) and post-harvest stages.

Cultivar	Ripening stage	Epicarp colour	DAB	Range I_AD_	Ethylene (nL h^-1^ g^-1^ FW)
Oro A	Pre-C	Yellow/green	99	1.50–2.00	0.00–1.11
	Post-C	Yellow/orange	105	0.00–0.06	4.30–44.71
	Post-H	Yellow/orange	107	< 0.05	40.33–150.25
Spring Crest	Pre-C	Yellow/green	79	0.80–1.00	0.21–1.17
	Post-C	Orange/red	91	0.00–0.10	3.55–13.32
	Post-H	Orange/red	93	< 0.05	35.77–89.88

Fruits were selected as much homogenous as possible within the most representative Index of Absorbance Difference (I_AD_) class for each stage. Ethylene production was measured in static condition sealing each fruit in a 1L jar.

DAB: days after bloom.

* significantly different (P<0.05).

In agreement with literature [2], MF fruits expressed a greater amount of endo-PG (increasing during ripening) than Oro A which showed a negligible enzyme content. The analysis of ethylene indicated that the climateric peak was reached faster in NMF than MF fruits and that, at this stage, the hormone production was higher in NMF. After two days from harvest, as expected [32], the ethylene production increased considerably in both cultivars reaching a higher value in Oro A fruits. No difference (P<0.05) in epidermal cell size was found between SC and Oro A (data not shown). Cuticle thickness did not change in both MF and NMF fruits during ripening and post-H, whereas the cell wall thickness increased from pre-C to post-C in Oro A and from post-C to post-H in SC.

Concerning water loss, on average, during the first two days of post-harvest, the percentage of fruit weight loss was significantly higher in SC than in Oro A, suggesting a greater loss of water in ripe MF fruits.

### Mechanical properties of peach fruits during ripening and post-harvest

The assessment of exocarp and mesocarp hardness/elasticity was made by a dynamometer which provided data on the capability of peach fruit tissues to face compression stress.

**Fig 2:**
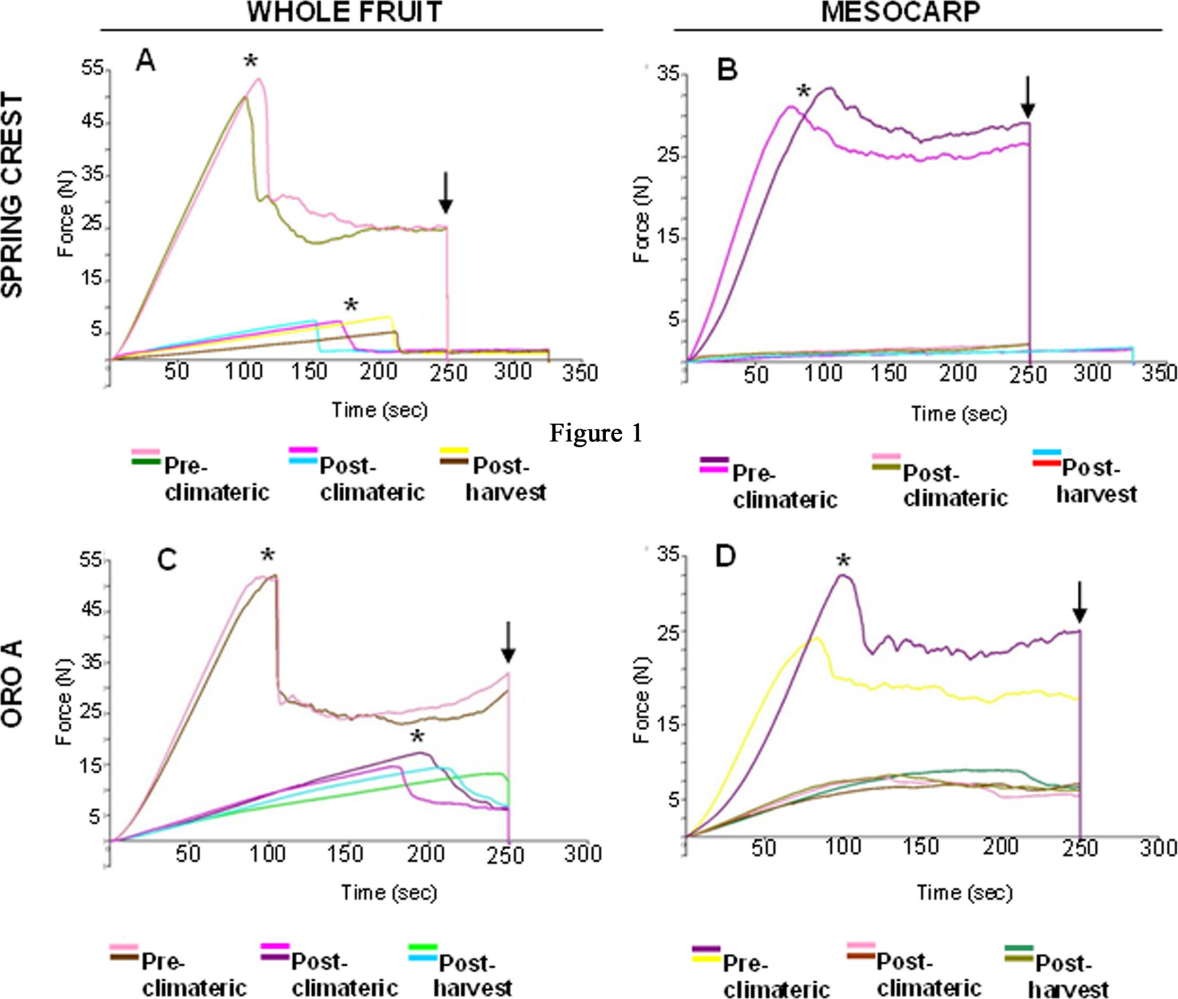
Example of cytograms showings curves obtained by dynamometer during peach fruit analysis. SC and Oro A whole (exocarp and mesocarp) and peeled (only mesocarp) fruits were analysed at pre-C, post-C and Post-H stage. Total energy values (mJ, arrow), which describes the energy required by the probe to penetrate all the tissues in a fixed time, breaking energy (*), the energy released during tissue cracking and Young’s modulus (YM), which defines the elastic characteristics of fruit, were recorded.

**Fig 3:**
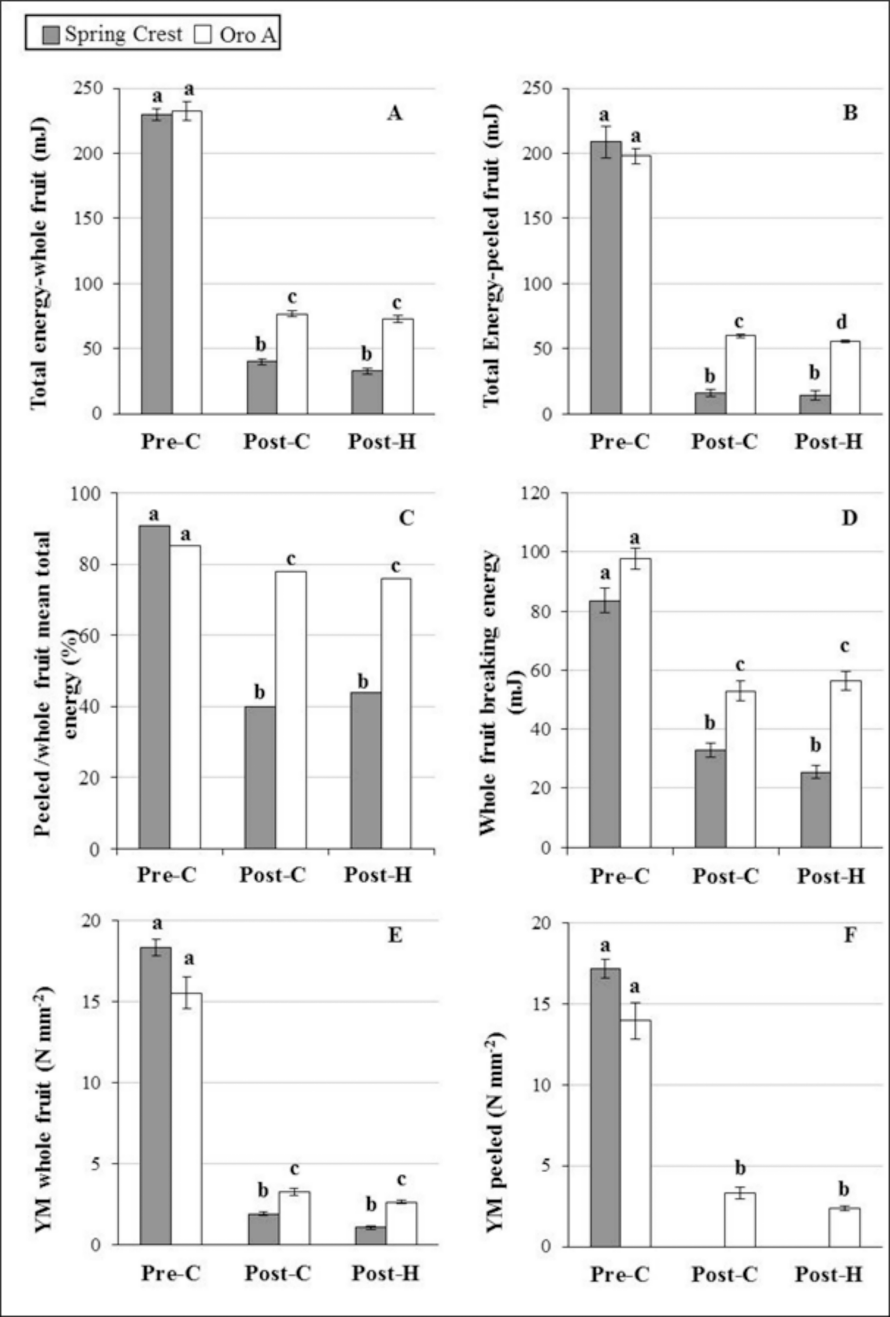
Mechanical properties of whole (exocarp and mesocarp) and peeled (only mesocarp) fruits during ripening and post-harvest. A and B, total energy (mJ); C, ratio between mean total energy values recorded in peeled and whole fruits (%); D, breaking energy (mJ); E and F, Young’s modulus (YM, (N/mm^2^). Different letters indicate significant differences (P<0.05).

By using a constant rate of probe penetration (0.04 mm/s) in a fixed time (250 s), dynamometer analysis provided different parameters which described fruit tissue behaviour during ripening. During probe penetration, epidermal/sub-epidermal tissues in the whole fruits and parenchymatic cells in peeled fruits underwent to deformation until the crack of their surface occurred. Subsequently, probe penetrated into the underlying mesocarp cells. Dynamometer recorded the total energy values (mJ; Fig 2, arrows and Fig 3A and 3B), which describes the energy required by the probe to penetrate all the tissues (epidermis and/or parenchyma) in a fixed time, the energy released during tissue cracking (breaking energy; Fig 2, asterisks and Fig 3D), and the Young’s modulus (YM) which defines the elastic characteristics of fruit: the higher the YM, the less elastic the tissue is (Fig 3E and 3F).

Specifically, the total energy value (mJ) includes the energy for tissue breaking (which depends on surface hardness/elasticity) and the energy for the following penetration into the tissues below the breaking point. In whole and peeled (mesocarp) fruits, no statistic difference was recorded between the mean energy values measured in pre-C Oro A and SC, suggesting that, before the ethylene production, NMF and MF fruits were equally firm. In both unripe MF and NMF fruits, mesocarp cells contributed considerably to the compression strain (about 85%; Fig 3C), suggesting a clear involvement of flesh in penetration resistance.

During ripening, a general decrease in the mean total energy was observed in both MF and NMF cultivars (Fig 2 and Fig 3A and 3B). This decrease was significantly more severe in SC than in Oro A cultivar (P<0.001) suggesting that, as expected, during maturation MF fruits softened more than NMF.

Between post-climateric and post-harvest stage, no significant total energy difference was observed in these 2 days of storage in both varieties (Fig 3A and 3B). In these last stages, the mesocarp contribution to penetration resistance was about 40% in SC whereas it was higher (75%) in Oro A (Fig 3C). Thus in Oro A, mesocarp continued to play an important role in maintaining firmness, whereas in SC its contribution decreased while exocarp played a prevalent role in compression strain.

Breaking energy and Young’s modulus (YM) were also considered to define the mechanical features of peach fruit tissues. Concerning breaking energy and time required to crack tissues of whole and peeled (mesocarp) fruits, no difference was observed between the two cultivars in pre-C phase (Fig 2, asterisks and Fig 3D). In the following stages, the NMF and MF whole fruits required a minor energy (Fig 2A and 2C and Fig 3D) and a longer time to be cracked (Fig 2A and 2C), indicating that during ripening both cultivars underwent softening. Nevertheless, in Oro A breaking energy was always significantly higher (p<0.01) than in SC confirming that NMF was more firm than MF fruit. In peeled fruits (mesocarp), breaking point was never reached in both cultivars in 250 seconds (Fig 2B and 2D) and dynamometer did not record breaking energy values indicating that the transition from elastic to plastic (viscoelastic) strain of tissues occurred. These data suggested a different capacity of mesocarp and exocarp tissues to withstand compression stress.

About YM, in both whole fruit and mesocarp, it was quantifiable only during the very early probe penetration since subsequently plastic strain prevailed over the elastic one (Fig 2). In pre-C phase, both MF and NMF fruits were stiff and showed a high YM which severely decreased during the following stages in parallel with softening (Fig 3E and 3F). However, YM remained higher in Oro A than in Spring Crest suggesting a greater stiffness of NMF tissues. Concerning the contribution of mesocarp and exocarp, in SC the whole fruit elasticity was due only to exocarp, as YM was never detected in mesocarp after ethylene development (Fig 3F) suggesting that flesh tissues underwent to viscoelastic strain. On the contrary in NMF fruits, both exocarp and mesocarp contributed to fruit elasticity as YM was detected also in mesocarp in post-C and post-H stages (Fig 2 and Fig 3E and 3F).

### Change in hydroxycinnamic acids and flavonoids content in NMF and MF peach fruits during ripening and post-harvest

The analysis of autofluorescence emitted from fresh fruit sections under UV excitation along with biochemical assays were performed to investigate polyphenol localization and content changes during ripening and post-harvest.

**Fig 4:**
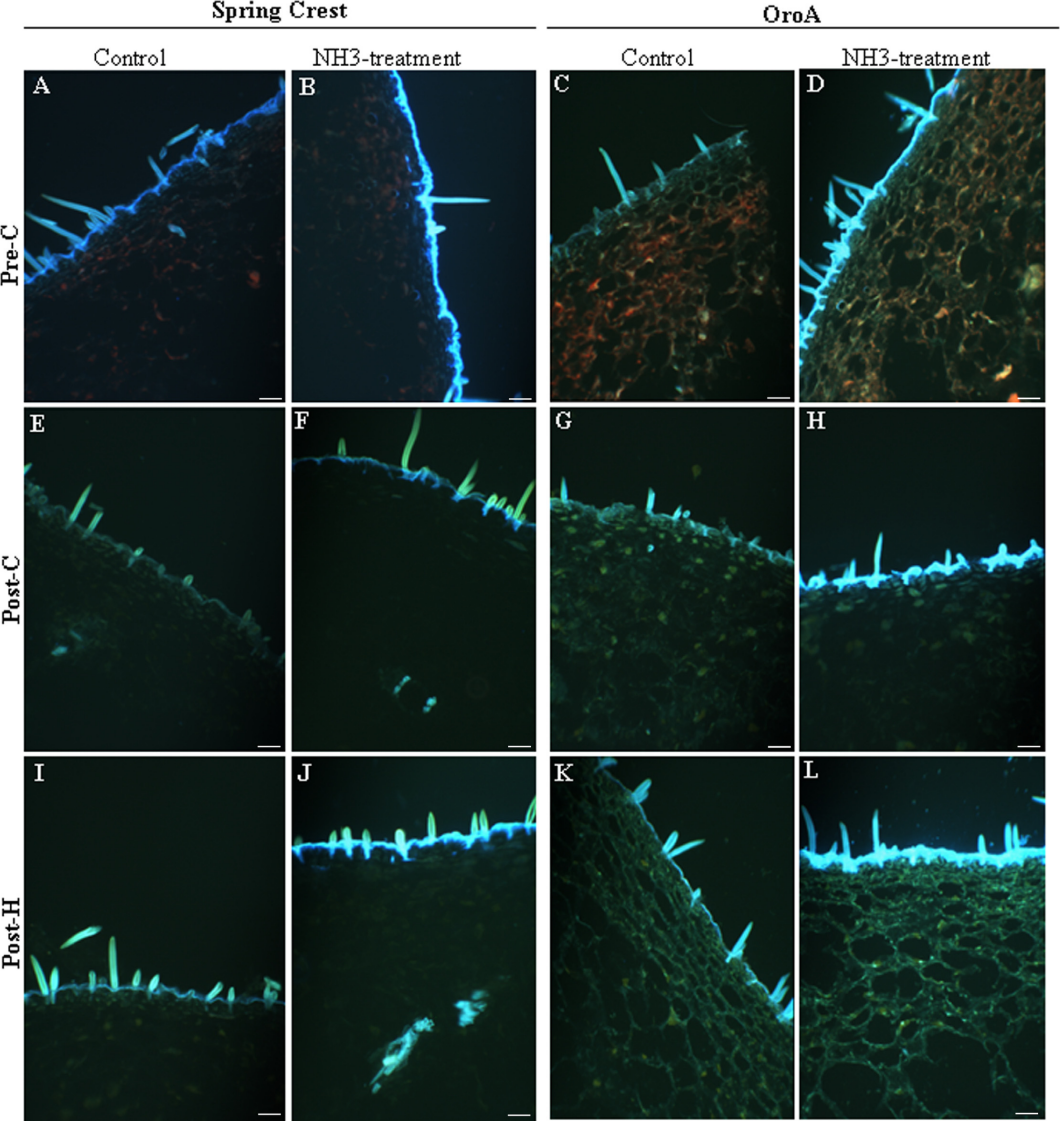
Representative pictures showing the localization of blue-green autofluorescence (BGF) emitted by HC and flavonoids in Oro A and SC exocarp sections before (control) and after ammonia treatment (NH_3_) during ripening and post harvest. Magnification bar: 50 μm.

**Fig 5:**
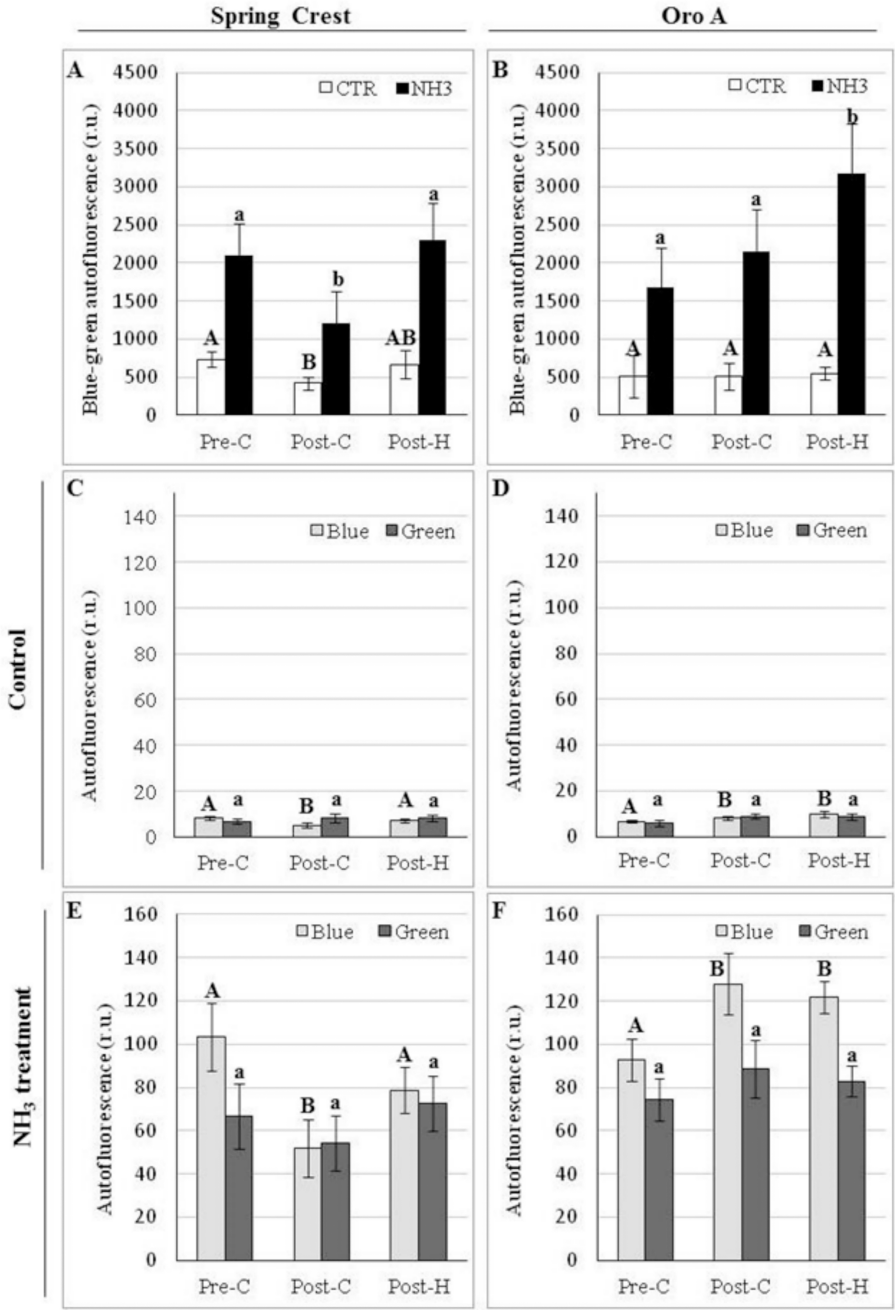
Image analysis quantification of changes in phenol-emitted BGF autofluorescence (A, B) and its blue and green components (C-F) in exocarp Oro A and SC sections during ripening and post-harvest. Different letters indicate significant differences (P<0.05) among the samples.

HC and flavonoids emitted blue/green autofluorescence (BGF) when excited by UV [20, 33–35]. NMF and MF exocarp and mesocarp were first analysed by traditional fluorescence microscope. Autofluorescence was observed mostly in epidermis cell walls, trichromes and vascular bundles, while it was very low in parenchymatic cells (Fig 4, control panels). On the basis of literature [20, 36, 22] blue autofluorescence in the xylem vessel wall was attributed to lignin, whereas BGF in epidermis cells could not be ascribed to a specific class of phenolic compounds (flavonoids or HC). During ripening, in SC exocarp, changes in polyphenolic compounds were observed in trichome and external/anticlinal cell wall and cuticle. Specifically image analysis revealed an autofluorescence decrease from pre-C to post-C phase and a subsequent increase after harvesting (Figs 4A, 4E, 4I and 5A). On the contrary in Oro A, autofluorescence tended to increase from pre-C to post-H (Figs 4C, 4G, 4K and 5B), although the increase was not significant.

**Fig 6:**
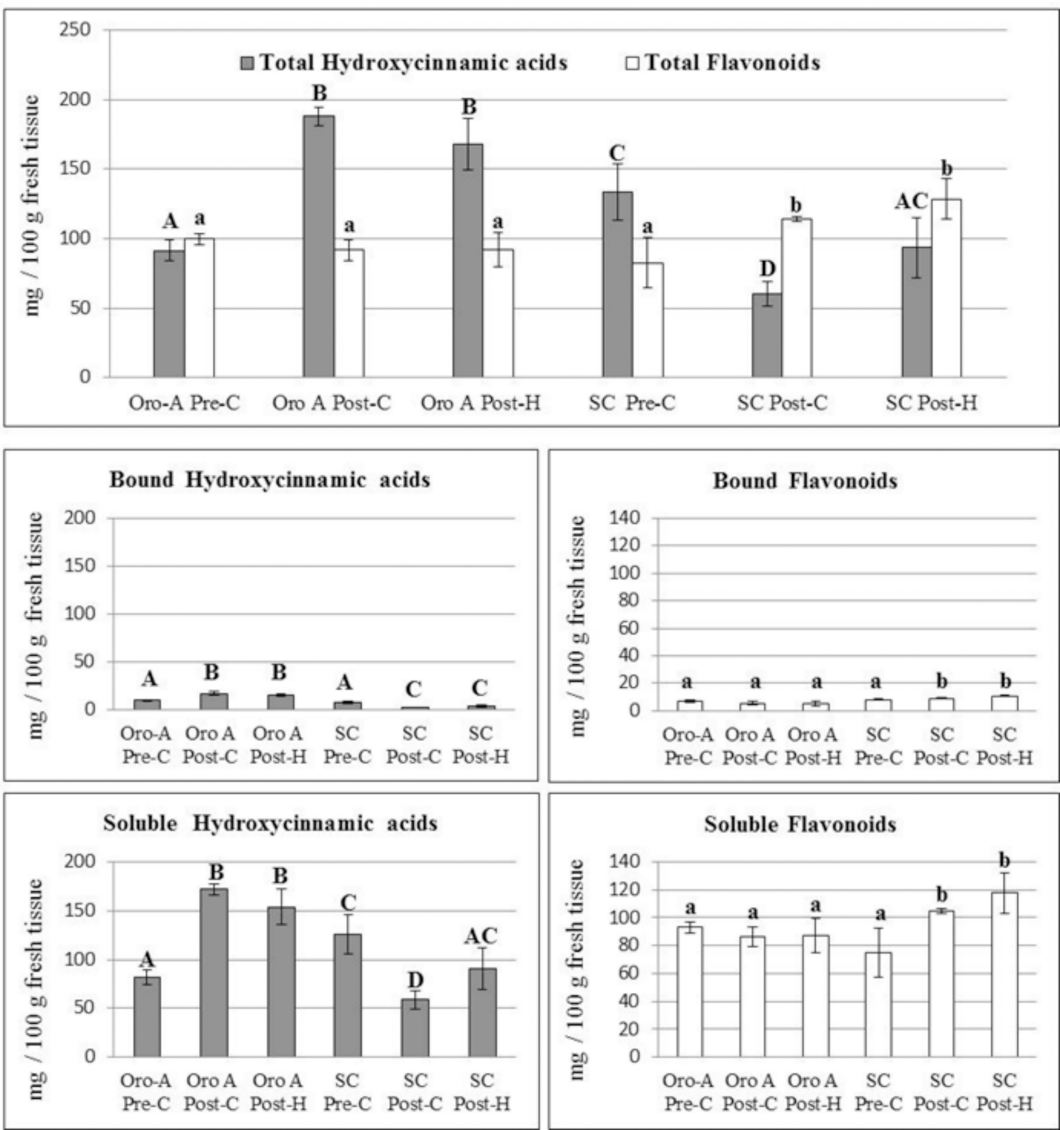
Biochemical quantification of total bound and soluble HC and flavonoids in Oro A and SC exocarp during ripening and post-harvest. Different letters indicate significant differences (P<0.05) among the samples.

In order to distinguish between the blue and green autofluorescence components of BGF, observed by traditional microscope and mainly emitted by HC and flavonoids, respectively [33], the sections were analysed with a confocal microscope. In SC and Oro A, both blue and green autofluorescence were mainly observed in epidermis external cell wall and trichomes at all the three stages analysed (data not shown). The quantification of the blue and green autofluorescence by image analysis showed that in SC the blue light followed the same pattern observed previously for BGF with a significant decrease (P<0.05) of autofluorescence intensity after climacteric peak and a significant increase (P<0.05) in post-H (Fig 5C). Green autofluorescence instead appeared to be constant during ripening and post-H (Fig 5C). Also in Oro A green autofluorescence was constant whereas the blue light tended to increase. It suggested that during ripening and post-H, HC trend was different in the two cultivars whereas the flavonoids’ one, emitting green light under UV excitation, did not significantly change. However biochemical analysis showed that during ripening the flavonoid content in exocarp was constant only in Oro A cultivar whereas it increased in Spring Crest, suggesting the presence of non-autofluorescent flavonoids in peach fruits (Fig 6). Specifically Fig 6 shows that biochemically quantified total HC trends were similar to those of the blue autofluorescence observed in Oro A and SC sections during ripening and post-H, whereas the trend of biochemically quantified total flavonoid was similar only to that of the green autofluorescence observed in Oro A, In SC while the green autofluorescence was constant, the biochemically quantified total flavonoid increased during ripening and remained higher in post-H. This result was also confirmed by the staining of flavonoids with diphenylboric acid 2-aminoethyl ester (NA) fluorochrome, whose fluorescence intensity increased in SC epidermal cell wall from pre to post-C stage (data not shown).

**Fig 7:**
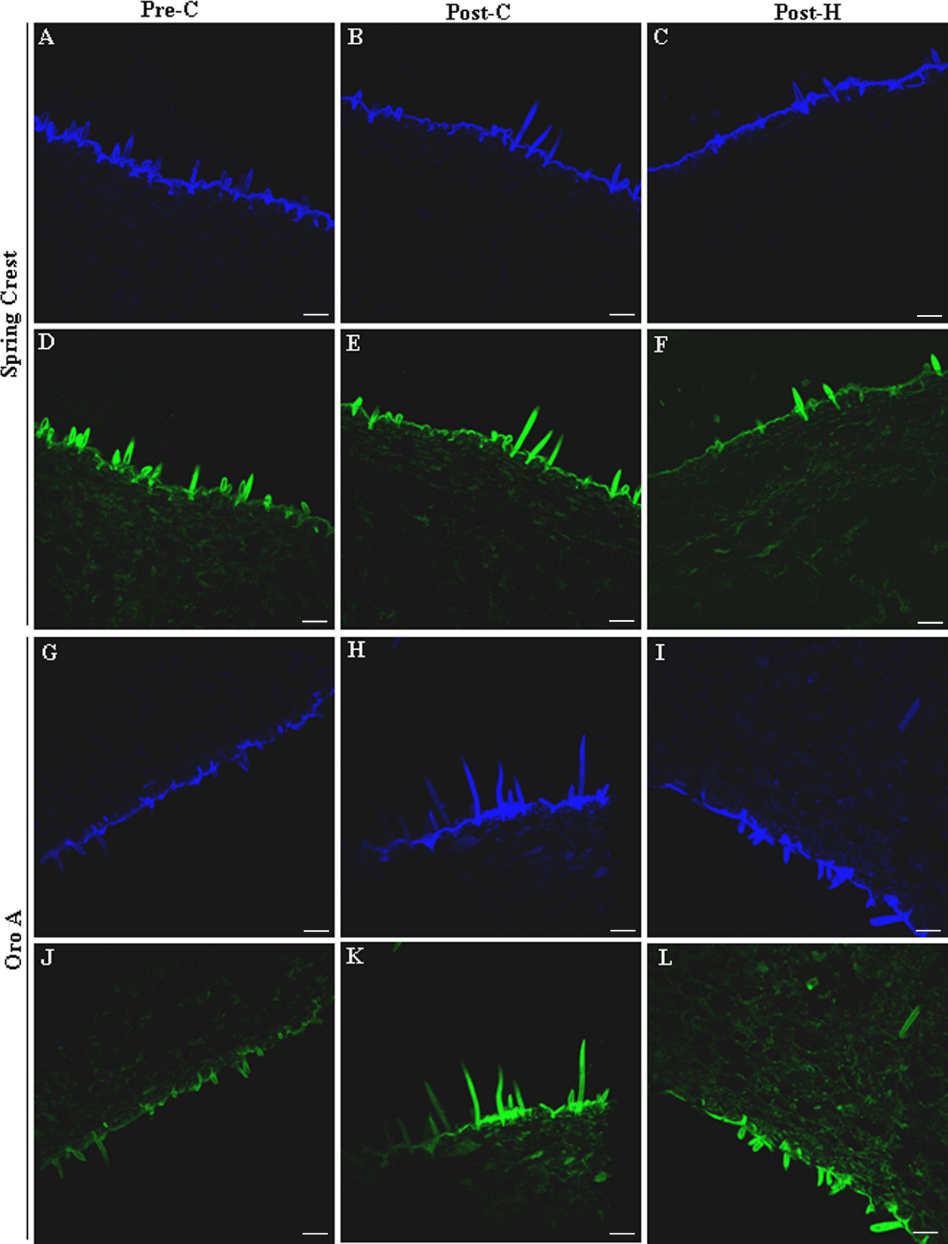
Representative pictures showing the localization of blue and green autofluorescence emitted by HC and flavonoids respectively, in Oro A and SC exocarp sections (after NH_3_ treatment) during ripening and post-harvest. Magnification bar: 50 μm.

To better study the variations in HC and flavonoid localization and content during ripening and post-H, an alkali treatment on fruit sections was also performed as it was demonstrated that NH_3_ treatment enhances BGF and induces the autofluorescence shift of esterified ferulic/coumaric acids from blue to green emission [35] (Fig 3, NH_3_ treatment panels). In both MF and NMF NH_3_-treated fruits, the BGF observed by traditional microscope was greatly enhanced but followed the same trend observed for untreated samples (control; Figs 4B, 4F, 4J and 5A and 5B). As for BGF, also the localization and the trends of blue and green autofluorescences observed by confocal microscope on both SC and Oro A NH_3_-treated sections were enhanced and very similar to those observed in untreated sections (Figs 5C–5F and 7). The localization and trend were similar probably because, being the amount of bound HC very low with respect to the total content (Fig 6), the autofluorescence shift from blue to green emission of these compounds was likely not enough to produce significant changes. Specifically Fig 6 shows that both bound HC and flavonoids were less than 10% out of the total polyphenolic compounds and that the trend of bound and soluble flavonoids was similar in MF and NMF fruits during ripening and post-H.

## Discussion

### Exocarp and mesocarp differently contributed to ripe MF and NMF fruit firmness

In melting and non-melting flesh peach cultivars, textural changes and firmness reduction seem to depend on different mechanisms involving wall modifications and cell turgor [2].

In order to define the contribution of cell wall modification and cell turgor to fruit softening, the firmness and the capability of fruit tissues to undergo the elastic/plastic strain were defined in Oro A (NMF) and Spring Crest (MF) cultivars by the use of a dynamometer. A set of parameters were recorded: (1) the total energy, describing the capability of fruit tissues to resist probe penetration, (2) the breaking energy, i.e. the energy required for cracking tissues, which is directly related to the hardness of tissues and (3) the Young’s modulus or coefficient of elasticity. All these parameters were recorded in whole and peeled fruits to better understand the contribution of exocarp and mesocarp tissues to the strain. Indeed in the whole fruits, total and breaking energy values were influenced by the strain capability of both exocarp (epidermis and cells immediately below) and mesocarp parenchymatic tissues. On the contrary, Young’s modulus was mostly dependent on the elasticity of the exocarp, as it was calculated before the breaking point. Dynamometer results showed that in pre/post-C and post-H stages, whole fruits of both cultivars underwent to a tissue crack during probe penetration. Breaking energy and total energy were similar in MF and NMF before climateric peak and decreased considerably later suggesting that in both fruits a loss of firmness occurred during softening. The loss of firmness was accompanied by a reduction of stiffness of tissues (YM value decreased) and by the transition from an elastic to a plastic pericarp strain. Although the general behaviour was similar in MF and NMF cultivars, significantly differences in breaking/total energy and YM values between the two fruit types were recorded. In SC, parameter values decreased much more than in Oro A confirming a more severe softening occurring in this cultivar. The analysis of peeled fruits allowed to understand the contribution of exocarp and mesocarp to this process. At pre-C stage, in both the cultivars, mesocarp mostly contributed to firmness (about 85–90%) because parenchymatic tissues showed cell wall integrity and turgid cells [2]. In fact before climacteric peak, softening physiological processes including the production of aromatic compounds and cell wall changes were not activated yet. Afterwards, as suggested by the peeled fruit/whole fruit total energy ratio (Fig 3C), Oro A firmness was dependent on both exocarp and mesocarp, whereas SC firmness mainly on exocarp.

Literature data suggest that cuticle and exocarp tissues can play a significant role in supporting mechanical stress. Biomechanical studies on isolated cuticle of cherry tomato fruits showed that cuticle could be considered as a layer that stiffens the cell wall, while epidermal and sub-epidermal cells gave strength to the cuticle [9, 14]. Thus, this important role of epidermis, sub-epidermic cells and cuticle would explain the significant contribution of exocarp in response to compression strain in both cultivars. On the other hand, only in MF fruits, mesocarp underwent a severe softening related to water loss and cell wall disassembly [2, 5, 37] explaining the observed negligible mesocarp contribution to Spring Crest firmness after climacteric stage.

The contribution of the sole exocarp to MF fruit stiffness was also indicated by the YM and breaking energy values. In MF fruit, YM and breaking energy were not detectable in the mesocarp after climacteric peak, suggesting a transition from an elastic to a plastic behaviour of flesh during softening. The transition should be related to the pectin modification and degradation occurring in cell wall. Pectin amount and pectin methylesterification level were considered the major contributor to wall elasticity [38, 39]. In *Arabidopsis*, lateral organ emergence in the shoot meristems correlated with an increase in tissue elasticity due to an increase in pectin de-methylesterification [40–42]. During fruits maturation, pectins are secreted in a highly methylesterified form and are selectively de-methylesterified [38]. In MF peach fruit, pectin methylesterase (PME) activity was higher at the beginning of softening phase supporting the endo-PG activity and inducing the following dramatic late softening of fruits [6]. Thus the degradation of pectins in MF during softening would be responsible of the transition from an elastic to a plastic behaviour of the mesocarp with a consequent loss of breaking energy and YM detection which would explain the principal contribution of exocarp to whole fruits stiffness.

In comparison with SC, Oro A whole fruit appeared significantly less elastic and more firm.

Because in tomato it has been observed that YM did not reflect the wall properties alone but also the turgor pressure of fruit cells [43], the integrity of cell wall and the presence of turgid cells should be both taken into account to explain the differences between MF and NMF peaches.

Concerning the cell turgor, in a previous work, we observed that during maturation, in Spring Crest all the pericarp cells lost turgidity whereas in Oro A the sub-epidermal cells remained turgid [2]. Moreover, in the present work, we showed that after harvesting water loss was higher in MF than in NMF fruits suggesting different transpiration properties of epidermis in the two ripe fruits. Thus the presence of turgid cells in NMF fruits could contribute to their higher firmness. In tomato, differences in firmness were ascribed only to differences in pericarp cell turgor since degradation of the primary wall and middle lamella in ripe fruits was observed not only in fruit that soften but also in mutants which maintain firmness [1]. However, in Oro A, endo-PG was not detected in pericarp cell wall and wall disassembly did not occurred [2] suggesting that the high contribution of parenchymatic cells to firmness of NMF fruits could be associated with cell wall integrity.

### Changes in biomechanical properties of exocarp are associated with changes in epidermal amounts of flavonoids and hydroxycinnamic acids

The epidermis and cuticle are thought to have an important influence on the biomechanical properties of ripening fruit [44–46]. Saladiè et al. [1] proposed a model in which the cuticle affects the softening of intact tomato fruit both directly, by providing a physical support, and indirectly, by regulating the water status. Among the components of epidermal cell wall, phenolic compounds such as flavonoids and HC were demonstrated to be tightly bound to the cutin, waxes and polysaccharides of different leaves and fruits [15, 20, 21, 47] and were indicated as important components affecting the properties of exocarp. In peach, Fernández and co-authors [22] reported that most cuticular phenolic compounds are often covalently bound to biopolymers, protecting fruits against an array of potential biotic and abiotic stress factors. The flavonoids, in particular, were widely studied and a very recent paper by España et al. [24] demonstrated that the alteration of flavonoids accumulation during tomato ripening affects cuticle wax accumulation, biomechanics, water permeability and molecular arrangement of cuticle components. At this regards in a different work España et al. [47] demonstrated that tomato ripening is accompanied by a depolymerization of the cutin matrix and an incorporation of phenolic compounds as fillers restraining the mobility of cutin chains. In our study, during softening, the content of flavonoids in SC epidermal cell wall/cuticle increased in parallel to a decrease of fruit firmness due to the loose of both cell turgor and adhesion. On the contrary in Oro A, epidermal cell wall flavonoids did not increase during softening. Similarly to “Delayed Fruit Deterioration” tomato cultivar [1], at ripe stage these NMF fruits were firmer and colorless as the CHS enzyme was undetectable in their exocarp and mesocarp (Fig B in S1 File).

Considering the data reported by Luque et al. [48], Dominguez et al. [15] and España et al. [24], we can speculate that the accumulation of flavonoids in epidermal cell wall/cuticle of peach can be related to the regulation of water transpiration. Specifically, in peach, the accumulation of flavonoids should sustain the water loss reducing the cell turgor and thus the fruit firmness. This hypothesis is also supported by the measurements of water loss we performed on ripe fruits during the two days after harvesting that showed a minor loss of water in NMF than in MF fruits containing a greater amount of flavonoids in epidermis cell wall.

Moreover, as suggested by previous works on tomato, flavonoids can also act as modulators in the cuticle mechanical properties conferring stiffness and reducing the cutin matrix deformation [24]. In peach, from pre-C to post-C stage, both MF and NMF softened. However in ripe MF fruit, firmness could be ascribed almost exclusively to the exocarp and in particular to cuticle, whereas in NMF the integrity of parenchymatic cell walls represented the major contribution. The greater amount of flavonoids in SC could be then related not only to water loss regulation but also to the need of this fruit to support the flesh by improving the cuticle stiffness. Also in softening tomato, it was suggested that cuticle becomes stiffer to compensate the loss of pericarp strength with a mechanical reinforcement mediated by flavonoids [14, 22, 45, 47].

However, tissues stiffness was also correlated with the presence of HC, which can bound cell wall components (polysaccharides, cutin and waxes) contributing to wall assembly, promoting tissue cohesion and restricting cell wall extensibility [17, 47]. In both Oro A and SC fruits most HC were localized in epidermal cell wall/cuticle as already reported for the peach cultivar Calrico [22]. From pre-C to post-C the content of HC significantly decreased in MF whereas increased in NMF, suggesting a role in maintaining fruit firmness for this class of polyphenols. On the other hand it should be also considered that the HC constituents of the epicuticular layer might regulate water transpiration maintaining turgid the epidermal and sub-epidermal cells as observed in ripe Oro A fruit [2, 20].

On the whole, we showed that the trend of HC and flavonoid content in epidermis cell wall/cuticle was different in NMF Oro A and MF SC fruit. Interestingly ripe soft SC peach contained a relative lower amount of HC and a higher amount of flavonoids, whereas ripe firmer Oro A peach a higher amount of HC and a lower amount of flavonoids. It suggests that, low levels of HC along with high level of flavonoids support water loss and fruit firmness reduction, whereas high level of HC along with lower level of flavonoids reduce transpiration and increase fruit firmness.

## Conclusion

The study of the mechanical properties of peach fruits during ripening and post-harvest demonstrated that the exocarp and mesocarp tissues differently contributed to MF and NMF fruit softening because they showed different mechanical characteristics that are the result of the interplay between diverse mechanisms. In SC, both the changes in cell wall structure and turgor induced a modification of tissue behaviour during softening. Parenchymatic mesocarp cells had a little role in the fruit reaction to compression and probe penetration response could be ascribed almost exclusively to the exocarp. The elasticity of exocarp was due to the presence of epidermis and cuticle which offered a mechanical resistance. In NMF fruits, cell wall disassembly did not occur and the loss of cell turgor was observed only in mesocarp and not in exocarp cells [2]. Thus, although the contribution of exocarp to firmness of the whole fruit was consistent, it was relatively lower than that of the mesocarp, suggesting that in addition to the cell turgor, the integrity of the cell wall played a key role in maintaining the NMF fruit firm. On the whole, conversely to what we hypothesized in our previous paper [2] with this investigation we demonstrated that the wall dismantling, through the endo-PG activity, contributes to the loss of MF fruit firmness. NMF fruit which does not contain endo-PG remains always more firm than MF fruit not only because it loses less water through the cuticle but also because it maintains cell wall integrity and adhesion. It derives that in peach the fruit firmness during softening process depends on the biomechanical features of exocarp and mesocarp, which can be modulated in different fruit types though different mechanisms mainly involving cell turgor and cell wall structure. Concerning exocarp the parallel investigation of polyphenols allowed us also to show that flavonoids and HC likely contribute to the characteristics of the fruit skin, modulating cell turgor and stiffness, as they are differently accumulated in the epidermis cell wall and cuticle of SC and Oro A fruits during ripening. Specifically we found an association between loss of cell turgor and presence of relative lower amount of HC and higher amount of flavonoids in epidermis cell wall/cuticle suggesting for peach a role of phenolic compounds in regulating the water status and the epidermis stiffness. Although further functional studies are needed to clarify the precise role played by the different classes of polyphenols, this work shows that phenols are important compounds for both peach and tomato softening. Nevertheless, differently from tomato, this work points out that the cell wall dismantling of pericarp cells is an additional mechanism of peach fruit softening which should be taken in consideration in ameliorating fruit quality and shelf life.

## Supporting Information

S1 File Fig A and Fig B. **Fig A.** Oro A (NMF) and Spring crest (MF) fruits at different stages of ripening. A and B: Oro A fruits at 99 and 105 DAB, respectively; C and D: Spring crest fruits at 79 and 91 DAB, respectively. Arrows: examples of pre-C and post-C fruits selected for the experiment on the basis of I_AD_. **Fig B.** Expression of CHS mRNA (A) and protein in ripening and 2-days-post-harvest ORO A and Spring Crest fruits. rCHS: recombinant CHS protein.
(DOCX)
