# Correction: Specific Changes of Exocarp and Mesocarp Occurring during Softening Differently Affect Firmness in Melting (MF) and Non Melting Flesh (NMF) Fruits

**DOI:** 10.1371/journal.pone.0147893

**Published:** 2016-01-22

**Authors:** The PLOS ONE Staff

## Notice of Republication

Figs 1–7 were published in error. The error was corrected in the HTML and PDF versions of this article on January 7, 2016, and the corrected figures are available in the supporting information of this article. The publisher apologizes for the errors. Please download this article again to view the correct version.

## Supporting Information

S1 Zip Corrected Figs 1–7. (ZIP)

## References

[1] Onelli E, Ghiani A, Gentili R, Serra S, Musacchi S, Citterio S (2015) Specific Changes of Exocarp and Mesocarp Occurring during Softening Differently Affect Firmness in Melting (MF) and Non Melting Flesh (NMF) Fruits. PLoS ONE 10(12): e0145341 doi: 10.1371/journal.pone.0145341 26709823 10.1371/journal.pone.0145341 PMC4692397

