# Supplementary material for: Specific Changes of Exocarp and Mesocarp Occurring during Softening Differently Affect Firmness in Melting (MF) and Non Melting Flesh (NMF) Fruits
Source: PLoS One. 2015 Dec 28;10(12):e0145341. doi: 10.1371/journal.pone.0145341 (PMC4692397; doi:10.1371/journal.pone.0145341)
Supplement: S1 File — Fig A. Oro A (NMF) and Spring crest (MF) fruits at different stages of ripening. A and B: Oro A fruits at 99 and 105 DAB, respectively; C and D: Spring crest fruits at 79 and 91 DAB, respectively. Arrows: examples of pre-C and post-C fruits selected for the experiment on the basis of IAD. Fig B. Expression of CHS mRNA (A) and protein in ripening and 2-days-post-harvest ORO A and Spring Crest fruits. rCHS: recombinant CHS protein. (DOCX) [file pone.0145341.s001.docx]

**Supporting Information**

**S1 File. Figure A and Figure B**


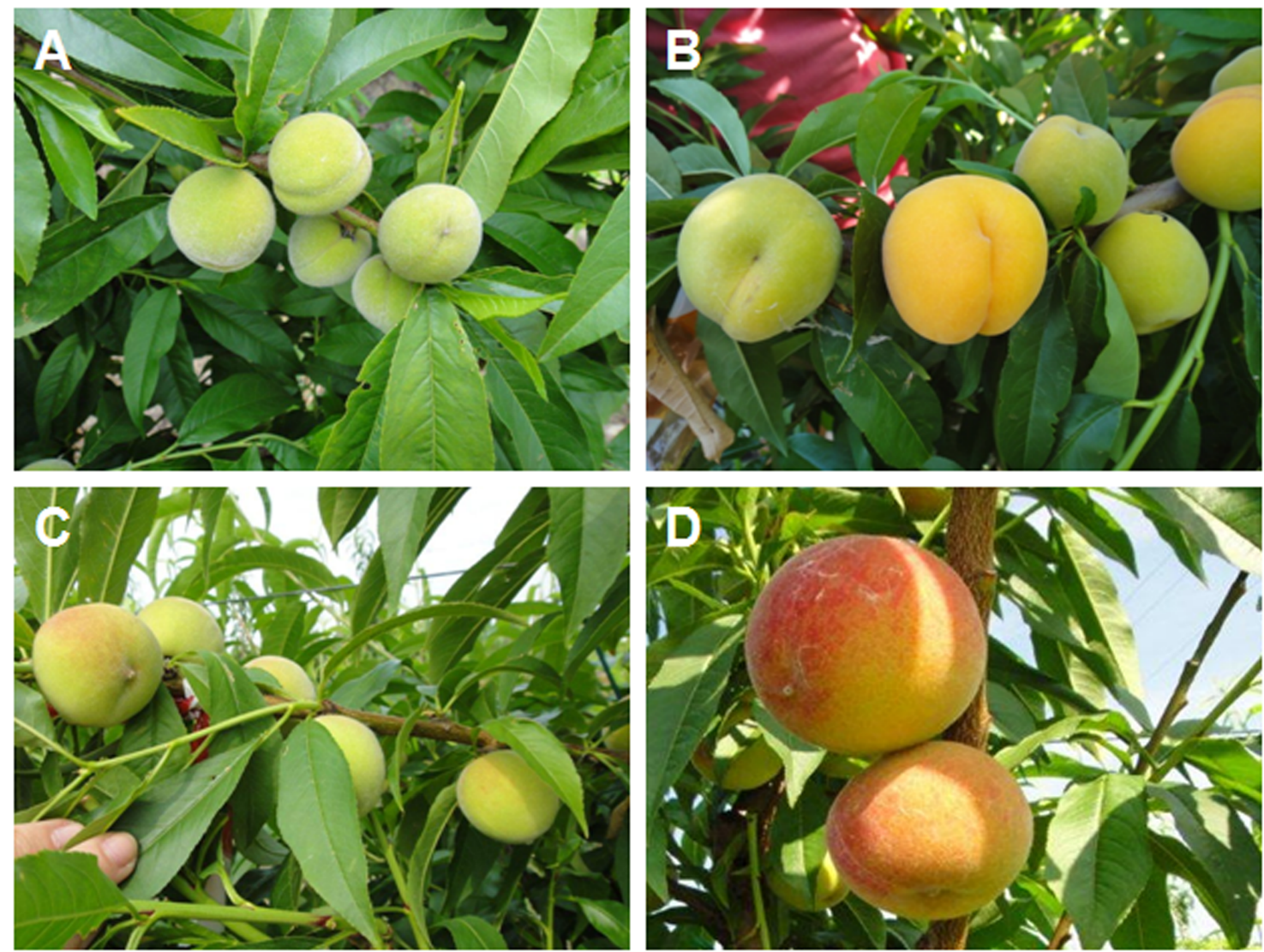


**Figure A** Oro A (NMF) and Spring crest (MF) fruits at different stages of ripening. A and B: Oro A fruits at 99 and 105 DAB, respectively; C and D: Spring crest fruits at 79 and 91 DAB, respectively.

Arrows: examples of pre-C and post-C fruits selected for the experiment on the basis of I_AD_.


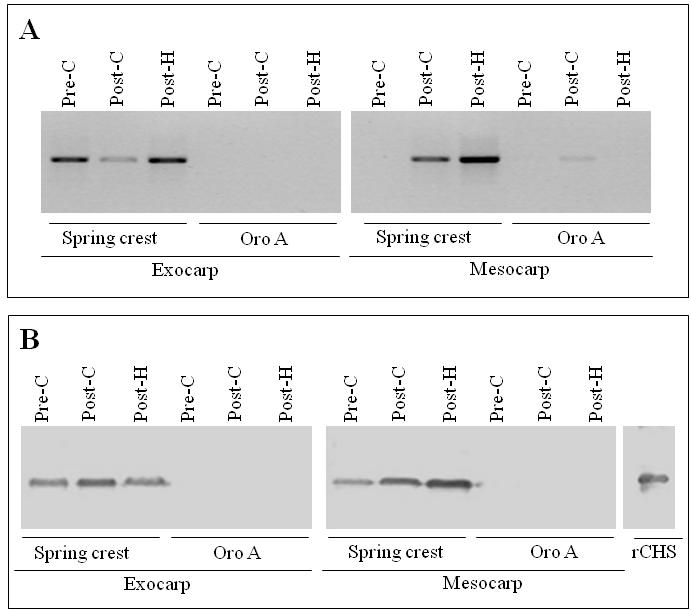


**Figure B** Expression of CHS mRNA (A) and protein in ripening and 2-days-post-harvest ORO A and Spring Crest fruits. rCHS: recombinant CHS protein.
