# Supplementary figures and images for: Correction: Specific Changes of Exocarp and Mesocarp Occurring during Softening Differently Affect Firmness in Melting (MF) and Non Melting Flesh (NMF) Fruits
Source: PLoS One. 2016 Jan 22;11(1):e0147893. doi: 10.1371/journal.pone.0147893 (PMC4723139; doi:10.1371/journal.pone.0147893)

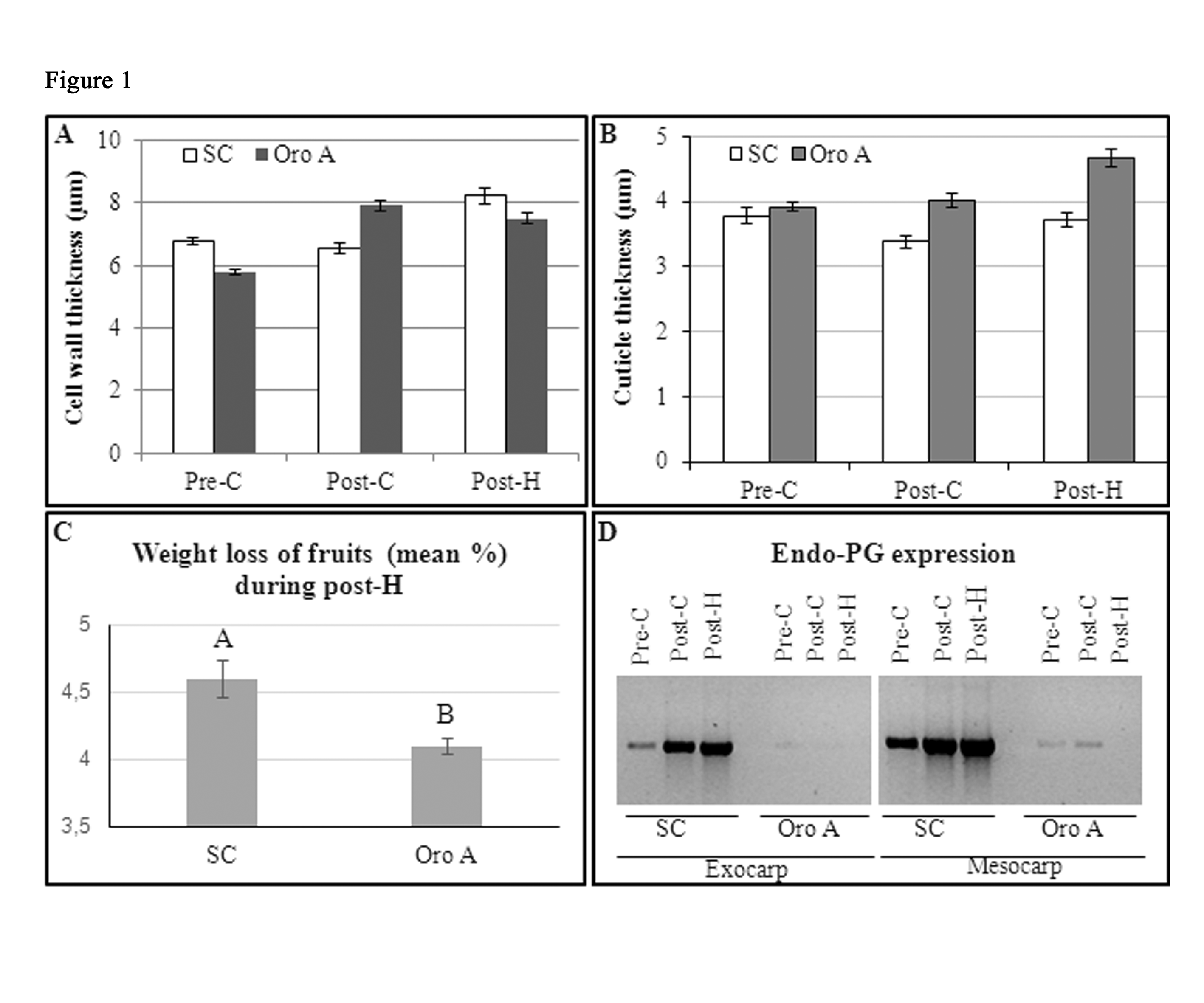

Supplement: S1 Zip — (ZIP) [file pone.0147893.s001.zip › Fig 1.tif]

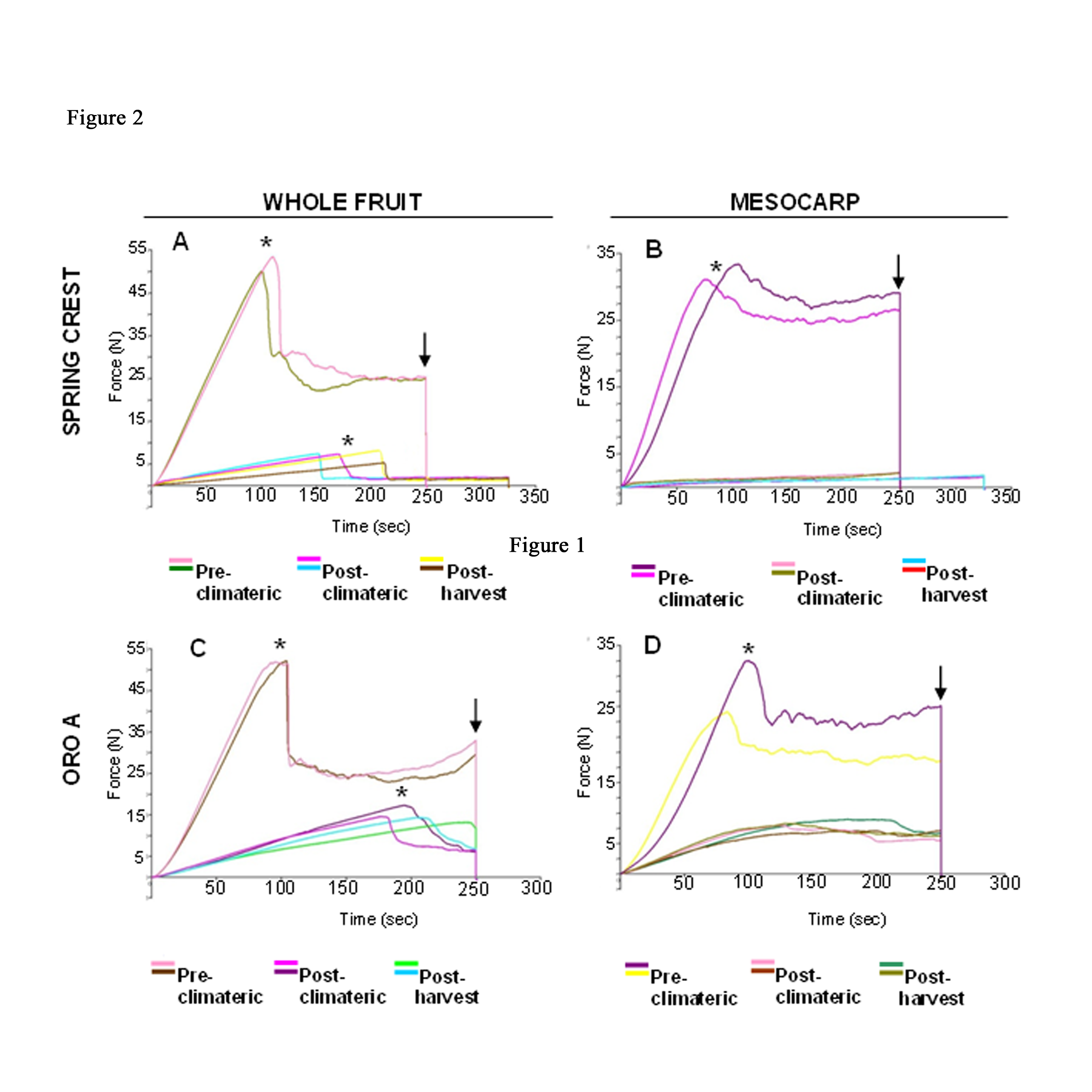

Supplement: S1 Zip — (ZIP) [file pone.0147893.s001.zip › Fig 2.tif]

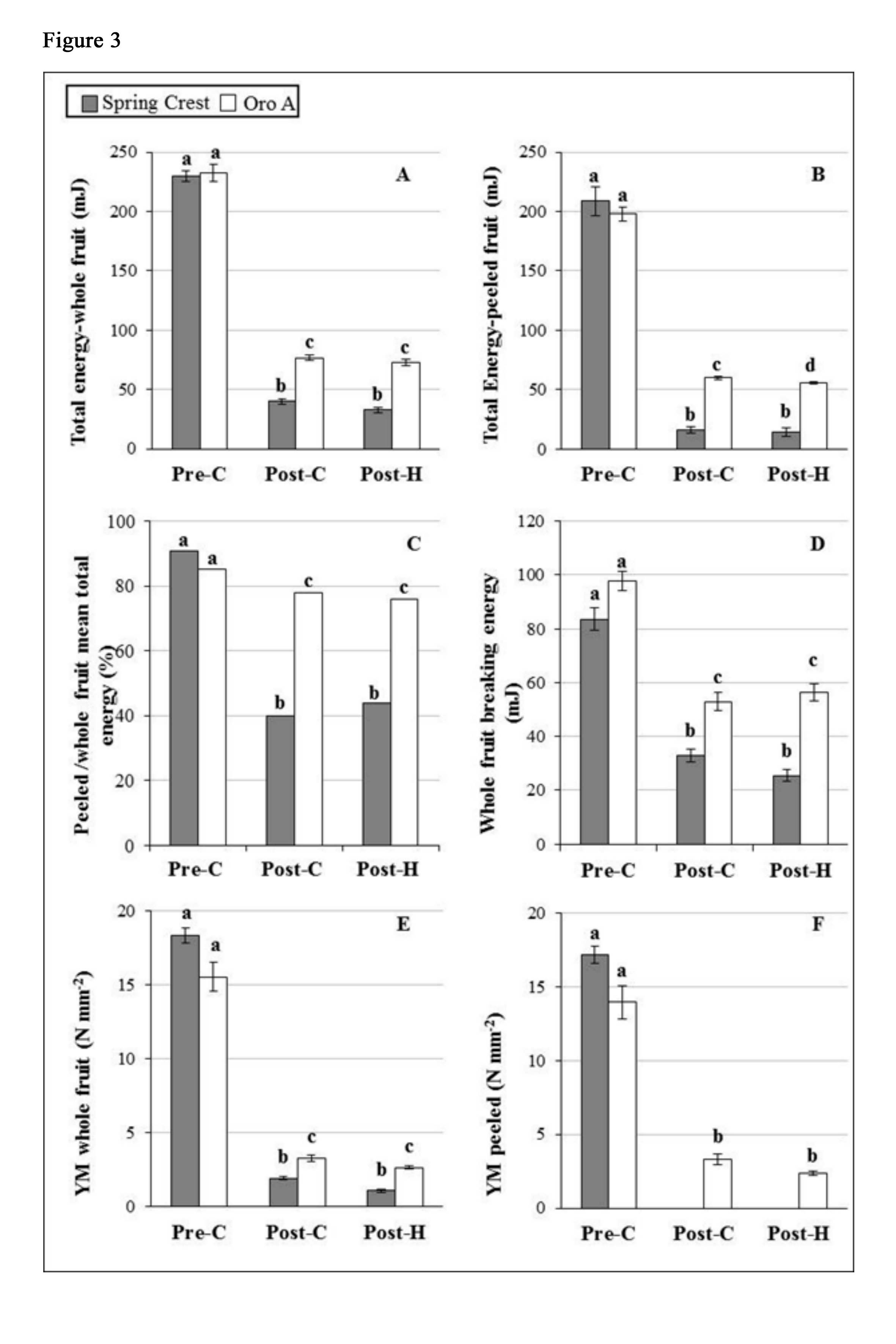

Supplement: S1 Zip — (ZIP) [file pone.0147893.s001.zip › FIG 3.TIF]

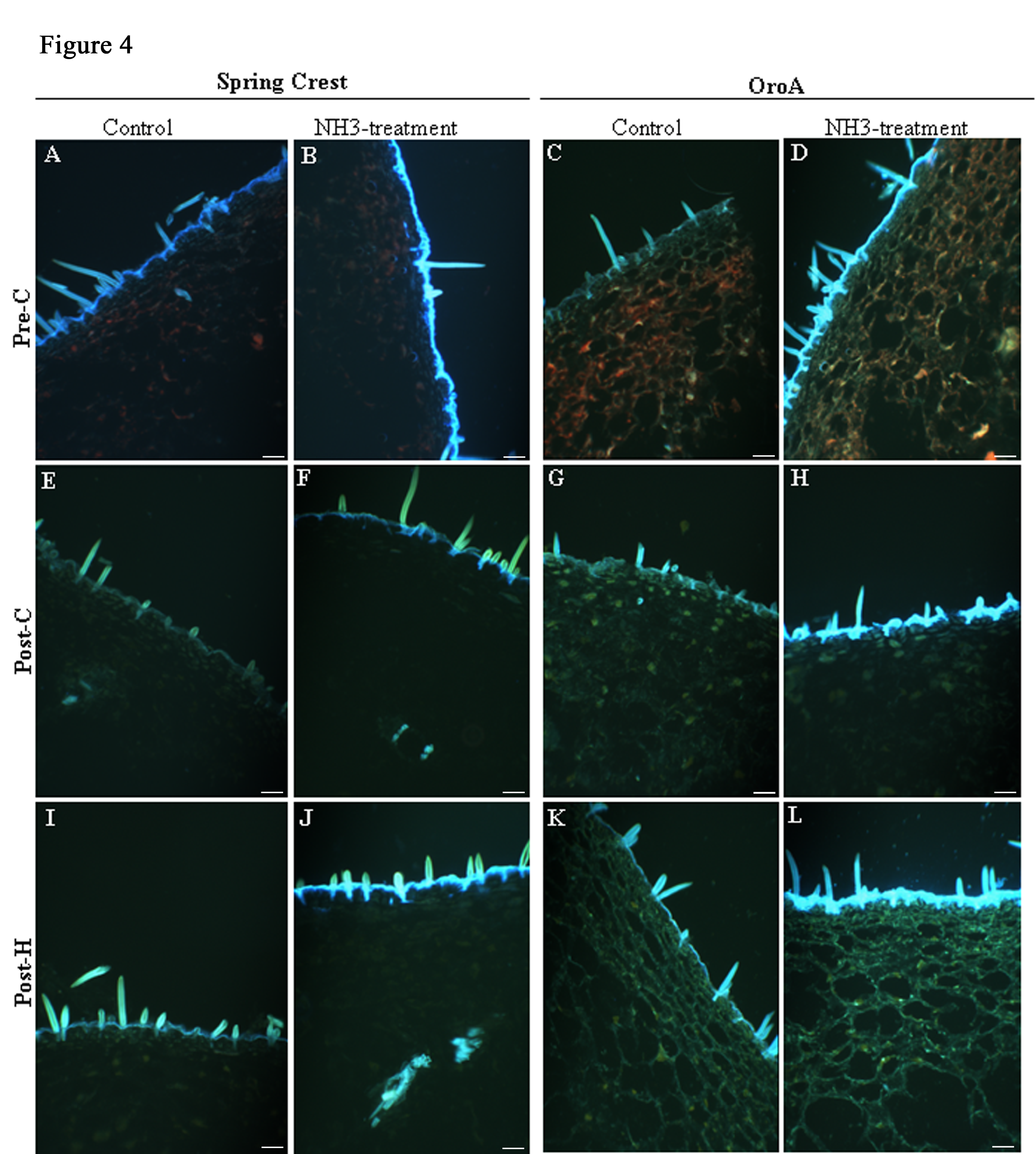

Supplement: S1 Zip — (ZIP) [file pone.0147893.s001.zip › Fig 4.TIF]

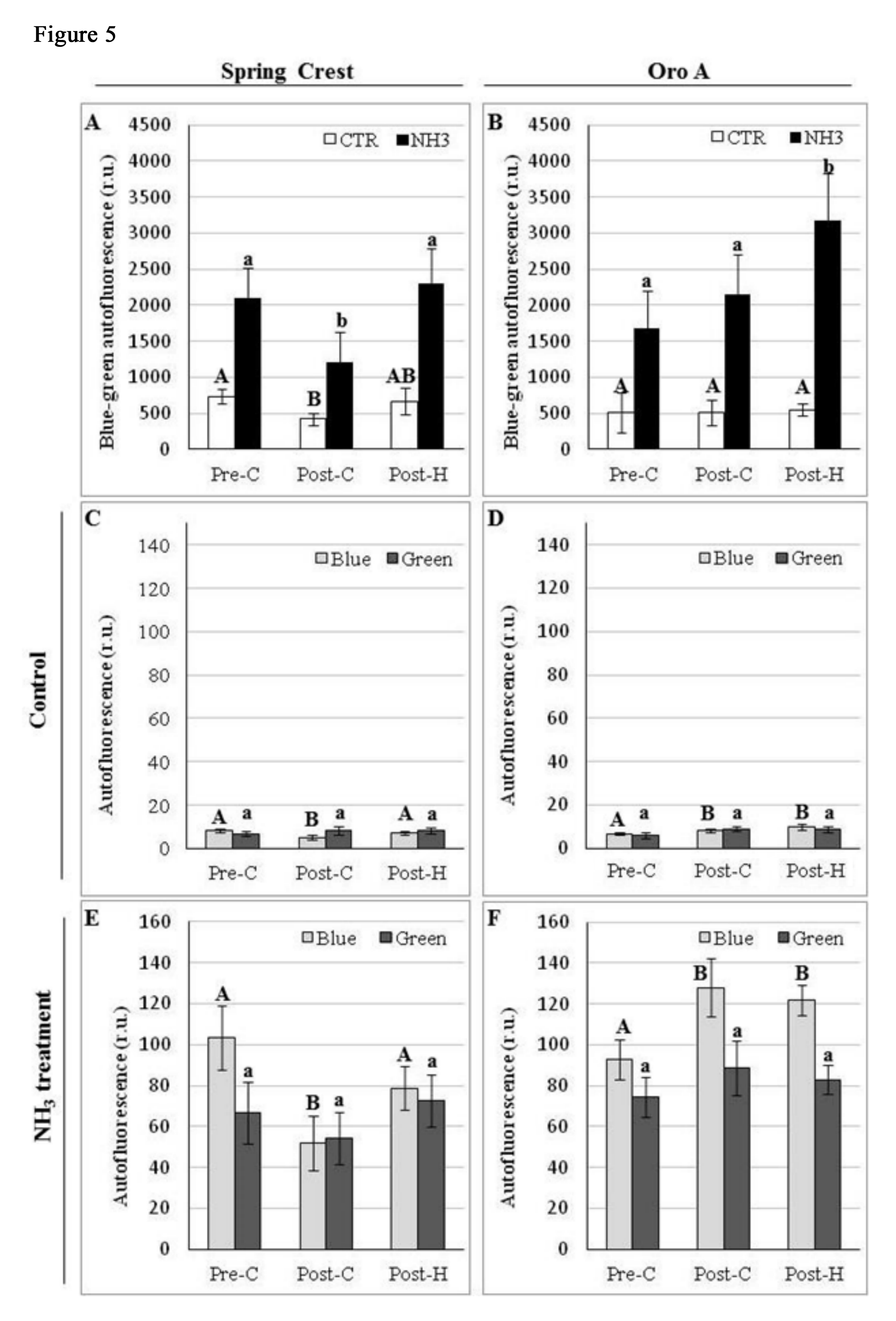

Supplement: S1 Zip — (ZIP) [file pone.0147893.s001.zip › Fig 5.TIF]

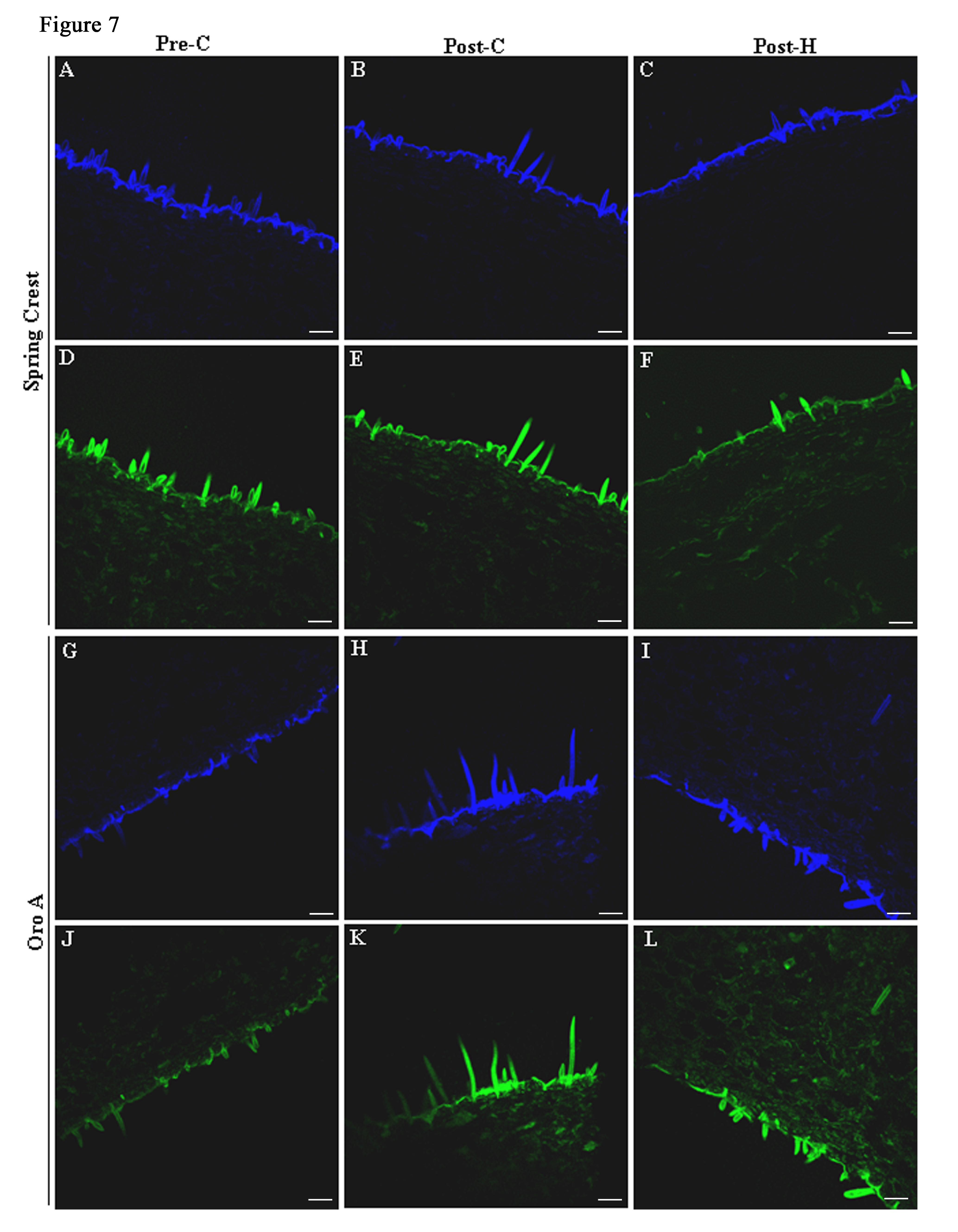

Supplement: S1 Zip — (ZIP) [file pone.0147893.s001.zip › Fig 7.tif]
